# Supplementary material for: Transdermal Maltose-Based Microneedle Patch as Adjunct to Enhance Topical Anesthetic before Intravenous Cannulation of Pediatric Thalassemic Patients Receiving Blood Transfusion: A Randomized Controlled Trial Protocol
Source: J Clin Med. 2022 Sep 8;11(18):5291. doi: 10.3390/jcm11185291 (PMC9501834; doi:10.3390/jcm11185291)
Supplement: Supplementary file 1 [file jcm-11-05291-s001.zip › jcm-1827318-supplementary.pdf]

Journal of
*Clinical Medicine*

MDPI

# SUPPLEMENTARY MATERIALS

S1: Patient information sheet and informed consent forms (a) English language, (b) *Bahasa Malaysia*

S2: Case report forms

**Material S1(a).** Patient information sheet and informed consent forms

## PATIENT INFORMATION SHEET

**Research Title:**

Transdermal Maltose-Based Microneedle Patch as Adjunct to Enhance Topical Anesthetic Before Intravenous Cannulation of Pediatric Thalassemic Patients Receiving Blood Transfusion: A Randomized Controlled Trial Protocol

**Introduction:**

You are invited to participate in a research study. Before participating in this study, it is crucial that you thoroughly read and understand the information provided in this sheet. However, before you take part or agree to continue in this research study, the study will be verbally explained to you, and you will be allowed to ask questions. After you are adequately satisfied that you understand this study and you wish to take part or continue to participate in this study, you must sign this informed consent form. You will be given a copy of this patient information sheet and consent forms to take home with you.

**Purpose of Study:**

Venipuncture (cannulation of blood vessel) elicits much pain and is regarded as the most frequently traumatic experience encountered among children. The pain may be reduced by applying topical anesthetic drugs. For example, EMLA® Cream (lidocaine 2.5% and prilocaine 2.5%) is a non-invasive and convenient means of administering anesthesia. The recommended duration of time taken for EMLA® Cream to work is around 60 minutes. However, in a busy clinical setting, the time is often expedited to 30 minutes. As such, microneedles had been introduced to enhance the delivery of topical anesthesia by puncturing the skin at a very superficial level to create multiple tiny tracts for faster action. The microneedle is a device containing multiple small needles made of sugar that will dissolve into the skin.

**What will the study involve?**

In this study, your child is about to undergo venipuncture for regular blood transfusion in 4 consecutive daycare visits. Depending on the sequence of interventions we have determined, your child will receive application of the topical anesthetic drug i.e., EMLA® Cream, with or without the introduction of the microneedle to the skin. This will result in numbness on the site of his/her hand intended for the blood transfusion, which then reduces pain. The incidence and severity of pain will be recorded after the cannula for blood transfusion has been inserted.

For this study, we would like to compare whether EMLA® Cream alone or in combination with microneedle results in better anesthetic effect. Therefore, there will be a maximum additional 30 minutes imposed to your child normal routine for this medicine to be applied, and for us to record the findings.

**Risks:**

This investigation poses minimal risk to participants and is unlikely to cause side effects. Nevertheless, the possible common side effects from the EMLA® Cream application include:

- pallor,
- redness,
- alterations in temperature sensation over the application area.

Additional risks associated with microneedle application might include possible mild-to-moderate skin irritation, especially in those with sensitive skin, such as

- redness,
- swelling,
- itching.

Should your child experience any problems, we will give your child medications to reduce the side effects of interventions accordingly.

**Benefits:**
There will be skin anesthesia effect from topical anesthetic drug, i.e., EMLA® Cream. Furthermore, the information from this study may add to the medical knowledge about the use of dissolvable solid maltose microneedle in transdermally delivering local anesthetic agents which may help future patients.

**Do you have to take part?**
Participation in this study is absolutely voluntary. Your child's medical care is not affected if you decide not to participate in this study. He/She will have the usual standard of care according to the daycare protocol.

If you agree to participate, you will be asked to sign the "Informed Consent Form". You will be given a copy of the informed consent form and this Patient Information Sheet. Should you decide to participate, you are still free to withdraw from the study at any time without giving a reason and penalty. If you decide to cease from participating in this study, you must inform your study investigator and no new data will be collected from your child. The researcher may also remove your child from the study for various reasons. In this event, your child will not lose his/her rights as a patient and will still receive the usual standard of care.

**Data & Confidentiality:**
Participant's confidentiality will be maintained throughout the investigation. The personal data will be anonymized. Hence your identity will be kept confidential. Data collected and entered into the Case Report Form remain the property of UKM. In the event of any publication regarding this study, your identity will remain confidential.

By signing the Informed Consent form attached, you (or your legally acceptable representative, if relevant) are authorizing such access to your study records.

**Payment and compensation:**
You do not have to pay, nor will you be paid to participate in this study. You do have to pay for the usual hospital charges.

**Whom can I ask about the study?**
If you have any questions about this study or your rights, please contact

| | |
|---|---|
| Principal Investigators: | Prof. Dr Cheah Fook Choe<br>Department of Paediatrics<br>UKM Medical Centre<br>Phone Number : 03-9145 5391/ 5380 |
| Co-investigator | Dr. Doris Lau Sie Chong<br>Department of Paediatrics<br>UKM Medical Centre<br>Phone Number : 03-9145 5387 |

**Signatures**

To be entered into this study, you or a legal representative must sign and date the signature page

Patient/Subject Information and Consent Form

(Signature Page)

***Research Title:*** Transdermal Maltose-Based Microneedle Patch as Adjunct to Enhance Topical Anesthetic Before Intravenous Cannulation of Pediatric Thalassemic Patients Receiving Blood Transfusion: A Randomized Controlled Trial Protocol

*Researcher's Name: Prof. Dr Cheah Fook Choe / Prof. Dr Azrul Azlan Hamzah*

To become a part of this study, you or your legal representative must sign this page. By signing this page, I am confirming the following:

- I have read all of the information in this Patient Information and Consent Form, including any information regarding the risk in this study and I, have had time to think about it.
- All of my questions have been answered to my satisfaction.
- I voluntarily agree to be part of this research study, to follow the study procedures, and to provide necessary information to the doctor, nurses, or other staff members, as requested.
- I may freely choose to stop being a part of this study at any time.
- I have received a copy of this Patient Information and Consent Form to keep for myself.

Patient Name (Print or type)

Patient Initials and Number

Patient I.C No. (New)

Signature of Parent or Legal Representative
(Add time if applicable)

Date (dd/MM/yy)

Name of Individual
Conducting Consent Discussion (Print or Type)

Signature of Individual
Conducting Consent Discussion

Date (dd/MM/yy)

Name & Signature of Witness

Date (dd/MM/yy)

Note: i) All subjects/patients who are involved in this study will not be covered by insurance.

**Material S1(b).** Patient information sheet and informed consent forms in local language

## BORANG MAKLUMAT DAN KEIZINAN PESAKIT

**Tajuk Kajian:**

Petak Jarum Mikro Maltosa Pada Kulit Bagi Mempertingkatkan Keberkesanan Ejen Bius Setempat Sebelum Penyisipan Saluran Intravena untuk Transfusi Darah Bagi Pesakit Talasemia Kanak-Kanak: Kajian Rawak Terkawal

**Pengenalan:**

Anda dijemput untuk menyertai satu kajian penyelidikan. Sebelum menyertai kajian ini, adalah penting untuk anda membaca dan memahami secara menyeluruh maklumat yang disediakan dalam lembaran ini.

Walau bagaimanapun, sebelum anda menyertai atau bersetuju untuk menyertai kajian penyelidikan ini, kajian ini akan dijelaskan secara lisan kepada anda dan anda akan diberi peluang untuk mengemukakan soalan. Setelah anda benar-benar berpuas hati bahawa anda telah memahami kajian ini, dan ingin menyertai atau terus mengambil bahagian dalam kajian ini, anda diwajibkan menandatangani borang persetujuan yang dikemukakan selepas ini. Anda akan diberikan salinan borang maklumat patient dan borang persetujuan untuk dibawa pulang bersama anda.

**Tujuan kajian:**

Tusukan vena sering menghasilkan kesakitan dan merupakan pengalaman yang traumatik yang sering dialami oleh kanak-kanak. Namun, kesakitan tusuk vena mampu dikurangkan dengan menyapu ubat bius setempat. Sebagai contoh, Krim EMLA (lidocaine 2.5% dan prilocaine 2.5%), adalah satu cara yang tidak invasif dan sesuai untuk pemberian ubat bius. Krim EMLA memerlukan jangka masa sekitar 60 minit untuk berkesan. Walaubagaimanapun, dalam persekitaran klinikal yang sibuk, tempoh masa sapuan krim EMLA selalunya dikurangkan kepada 30 minit. Oleh itu, jarum mikro telah diperkenalkan untuk meningkatkan keberkesanan ubat bius sapuan dengan cara cucukan pada paras permukaan kulit bagi tujuan menghasilkan saluran kecil untuk membolehkan ubat bius topikal diserap dengan lebih cepat. Jarum mikro merupakan suatu peralatan yang mengandungi jarum kecil diperbuat daripada gula yang akan diserap oleh kulit.

**Bagaimana kajian ini dijalankan?**

Untuk kajian ini, anak anda akan menerima tusukan vena untuk pemindahan darah secara berkala melibatkan 4 kunjungan berturut-turut ke pusat rawatan harian. Bergantung kepada turutan intervensi yang telah kami kenal pasti, anak anda akan menerima sapuan ubat bius setempat, Krim EMLA, dengan atau tanpa tusukan jarum mikro pada kulit. Ini akan menyebabkan rasa kebas pada kawasan tangan yang telah ditetapkan untuk tujuan pemindahan darah yang seterusnya akan mengurangkan rasa sakit. Insiden dan keterukan kesakitan akan direkodkan selepas kanula untuk tujuan pemindahan darah telah selesai dimasukkan.

Untuk kajian ini, kami berhasrat untuk membandingkan sama ada penggunaan jarum mikro tanpa atau dengan kombinasi Krim EMLA akan menghasilkan kesan bius yang lebih baik. Oleh itu, tambahan masa maksimum selama 30 minit pada rutin lawatan yang biasa anak anda lalui bagi tujuan pemberian ubat dan merekod dapatan kajian.

**Risiko kajian ini:**

Penyelidikan ini membabitkan risiko yang minimum kepada peserta dan mempunyai kemungkinan yang rendah

untuk menyebabkan kesan sampingan. Walaubagaimanapun,antara kesan sampingan yang mungkin berlaku akibat aplikasi krim EMLA adalah merangkumi

- kepucatan,
- kemerahan,
- perubahan suhu di kawasan aplikasi.

Risiko tambahan yang disebabkan oleh aplikasi jarum mikro, terutamanya bagi peserta yang memiliki kulit sensitive, berkemungkinan akan mengalami kerengsaan kulit ringan hingga sederhana seperti

- kemerahan,
- bengkak,
- gatal,

Sekiranya anak anda mengalami sebarang masalah, kami akan memberi ubat kepada anak anda untuk mengurangkan kesan sampingan intervensi.

**Faedah kajian ini:**
Aplikasi krim EMLA dan jarum mikro dipercayai dapat membantu mengurangkan kesakitan semasa tusukan vena untuk pemindahan darah. Selain itu, maklumat dari kajian ini dapat menambahkan lagi pengetahuan perubatan mengenai penggunaan jarum mikro maltosa yang terlarut sebagai sistem penghantaran transdermal untuk ejen bius setempat agar dapat membantu pesakit lain pada masa hadapan.

**Adakah anda perlu menyertai kajian ini?**
Penyertaan anda di dalam kajian ini adalah secara sukarela. Rawatan perubatan untuk anak anda tidak terjejas sekiranya anda memutuskan untuk tidak mengambil bahagian dalam kajian ini. Anak anda masih akan mendapat rawatan seperti biasa dan sama mengikut protokol pusat jagaan harian.

Sekiranya anda bersetuju untuk menyertai kajian ini, anda akan diminta untuk menandatangani "Borang Persetujuan Termaklum". Anda akan diberikan satu salinan borang persetujuan termaklum dan lembaran maklumat pesakit ini. Sekiranya anda bersetuju untuk mengambil bahagian, anda masih bebas untuk menarik diri dari kajian ini pada bila-bila masa tanpa sebarang alasan dan tanpa dikenakan penalti. Sekiranya anda memutuskan untuk menarik diri daripada kajian ini, anda perlu memaklumkan penyelidik kajian ini dan seterusnya tiada data baru akan diambil daripada anak anda.

Penyelidik juga mungkin akan mengeluarkan anak anda dari kajian ini atas sebab-sebab tertentu. Sekiranya keadaan ini berlaku, anak anda tidak akan kehilangan haknya sebagai pesakit dan akan terus menerima rawatan penjagaan seperti mana kebiasannya.

**Data & Kerahsiaan:**
Kerahsiaan peserta akan dilindungi sepanjang penyelidikan. Data peribadi akan dirahsiakan. Oleh itu identiti anda akan disimpan secara sulit. Data yang dikumpulkan dan dimasukkan dalam Borang Laporan Kes akan kekal menjadi hak milik UKM. Sekiranya terdapat penerbitan mengenai kajian ini, identiti anda akan kekal dirahsiakan.

Dengan menandatangani borang persetujuan yang dilampirkan sekali, anda (atau wakil anda yang boleh diterima secara sah, jika relevan) membenarkan akses untuk rekod kajian anda.

**Bayaran & Pampasan:**
Anda tidak perlu membayar ataupun akan menerima sebarang bayaran untuk penyertaan dalam kajian ini. Anda masih perlu membayar untuk perkhidmatan hospital seperti biasa.

**Siapakah yang boleh anda hubungi untuk maklumat lanjut?**
Sekiranya anda mempunyai sebarang kemusykilan, berkenaan kajian penyelidikan ini atau hak-hak anda, sila hubungi

| | |
|---|---|
| Penyelidik utama: | Prof. Dr Cheah Fook Choe<br>Jabatan Pediatrik<br>Pusat Perubatan Universiti Kebangsaan Malaysia<br>No. Telefon : 03-9145 5391/ 5380 |
| Penyelidik Bersama: | Dr. Doris Lau Sie Chong<br>Jabatan Pediatrik<br>Pusat Perubatan Universiti Kebangsaan Malaysia<br>No. Telefon : 03-9145 5387 |

**Tandatangan**
Untuk menyertai kajian ini, anda atau wakil anda yang sah mesti menandatangani halaman tandatangan berserta tarikh.

Borang Maklumat Pesakit/Subjek dan Persetujuan
(Halaman Tandatangan)

***Tajuk Penyelidikan:*** Petak Jarum Mikro Maltosa Pada Kulit Bagi Mempertingkatkan Keberkesanan Ejen Bius Setempat Sebelum Penyisipan Saluran Intravena untuk Transfusi Darah Bagi Pesakit Talasemia Kanak-Kanak: Kajian Rawak Terkawal

*Nama penyelidik: Prof. Dr Cheah Fook Choe / Prof. Dr Azrul Azlan Hamzah*

Untuk menyertai kajian ini, anda atau wakil sah anda mesti menandatangani halaman ini. Dengan menandatangani halaman ini, saya mengesahkan yang berikut:

- Saya telah membaca semua maklumat dalam Borang Maklumat Pesakit dan Persetujuan ini termasuk mana-mana maklumat berkaitan dengan risiko dalam kajian ini dan saya diberi masa untuk memikirkan tentangnya.
- Semua soalan saya telah dijawab sehingga saya berpuas hati dengan jawapannya.

- Saya secara sukarela bersetuju untuk menyertai kajian penyelidikan ini, untuk mengikuti prosedur kajian, dan memberi maklumat yang diperlukan kepada doktor, jururawat, atau ahli kakitangan lain, seperti yang diminta.
- Saya boleh bebas memilih untuk berhenti daripada kajian ini pada bila-bila masa.
- Saya telah menerima satu salinan Borang Maklumat Pesakit dan Persetujuan untuk simpanan saya.

________________________________ ____________________

Nama Pesakit (Tulis atau taip) Nama ringkas dan Nombor Pesakit

________________________________

K.P Pesakit No. (Baru)

________________________________ ____________________

Tandatangan Waris atau Wakil Sah Tarikh (dd/MM/yy)
(Tambah waktu, jika berkenaan)

________________________________

Nama Individu
Menjalankan Perbincangan Persetujuan (Tulis atau Taip)

________________________________ ____________________

Tandatangan Individu Tarikh (dd/MM/yy)
Menjalankan Perbincangan Persetujuan

________________________________ ____________________

Nama & Tandatangan Saksi Tarikh (dd/MM/yy)

<u>Nota:</u> i) Semua subjek/pesakit yang terlibat dalam kajian ini tidak akan dilindungi oleh insurans.

**Material S2.** Case report forms

**CASE REPORT FORM (CRF)**

**Research title: Transdermal Maltose-Based Microneedle Patch as Adjunct to Enhance Topical Anesthetic Before Intravenous Cannulation of Pediatric Thalassemic Patients Receiving Blood Transfusion: A Randomized Controlled Trial Protocol**

**Study Investigators:** 1) Prof. Dr Cheah Fook Choe

2) Prof. Dr Azrul Azlan Hamzah

**Nama ringkas:** *Subject Initials* ☐☐☐ **ID Subjek:** *Subject ID* ☐☐☐☐☐ **Tarikh:** *Date* ☐☐ / ☐☐ / ☐☐

Hari *Day* Bulan *Month* Tahun *Year*

**Kod Rawak:**
*Randomization Code*

1. ***Demografi*** *Demography*

Nama Subjek *Subject's Name* : ____________________________________________

Usia / *Age* : ______________(tahun *years*) ______________(bulan *months*)

No. Pend / *RN* : ____________________

No. Tel : (H.)____________________ (H/P) ________________

Tarikh lahir *Date of birth* : ________________/________________/ ________________
Hari dd Bulan mm Tahun yyyy

Tarikh keizinan *Date of consent* : ________________/________________/ ________________
Hari dd Bulan mm Tahun yyyy

**Jantina** *Gender* : ☐ Lelaki *Male* ☐ Perempuan *Female*

**Kaum / Etnik** *Race / Ethnicity* : ☐ Melayu *Malay* ☐ India *Indian*
☐ Cina *Chinese* ☐ Lain-lain, sila nyatakan: __________ *Other (Please specify)*

2. **Sejarah Penyakit Keluarga**
   *Family history* of illnesses

| **Darjah Pertama** *First Degree* | Ya *Yes* | Tidak *No* | **Darjah Kedua** *Second Degree* | Ya *Yes* | Tidak *No* |
|---|---|---|---|---|---|
| Ibu *Mother* | ☐ | ☐ | Pakcik *Uncle* | ☐ | ☐ |
| Bapa *Father* | ☐ | ☐ | Makcik *Aunt* | ☐ | ☐ |
| Adik-beradik *Siblings* | ☐ | ☐ | Sepupu *Cousins* | ☐ | ☐ |
| | | | Datuk-Nenek *Grandparent(s)* | ☐ | ☐ |

Sila nyatakan jenis penyakit dan umur ketika diagnosis:
*Please specify types of illnesses and age at diagnosis:*

## 3. KAJIAN SEJARAH TALASEMIA / PENGAMBILAN UBATBERKAITAN

*Review of Relevant History of Thalassemia / Medications for Thalassemia*

**Sejarah Perubatan / Pengambilan Ubat Berkaitan Talasemia**
*Relevant Medical / Drug History related to Thalassemia*

**Penemuan klinikal yang penting**
*Clinically-significant findings*

**Later belakang penyakit** *Disease background*

Diagnosis :
*Diagnosis*

- ☐ Talasemia bergantung kepada transfusi darah *Transfusional Dependant Thalassemia (TDT)*
- ☐ Talasemia tidak bergantung kepada transfusi darah *Non-Transfusional Dependant Thalassemia (NTDT)*

Sila nyatakan :
*Please specify*

- ☐ Thalassemia Major
- ☐ Hb E Beta Thalassemia
- ☐ Hb H Constant Spring
- ☐ Lain-lain, sila nyatakan: ______________________________ *Others (Please specify)*

Usia diagnosis :
*Age at diagnosis*

Usia permulaan transfusi :
*Age at initial transfusion*

Kekerapan semasa :
*Current frequency*

**Terapi kelasi zat besi** *Iron chelation therapy*

Usia permulaan terapi pengelatan :
*Age at initial chelation therapy:*

Pilihan terapi kelasi zat besi :
*Iron chelation therapy*

- ☐ Desferrioxamine (DFO)
- ☐ Deferiprone (DFP)
- ☐ Deferasirox (DFX)

Dos ubatan:
*Dosage*

## 4. KAJIAN SEJARAH PERUBATAN / PENGAMBILAN UBATBERKAITAN

*Review Relevant Medical History / Medications*

**Sejarah Perubatan**
*Relevant Medical History*

**Pengambilan Ubat Berkaitan (nyatakan nama ubat, tujuan pemberian, cara pemberian, kekerapan dan dos harian**
*Medications (State name, indications, administration route, frequency, and daily dose)*

**Penemuan klinikal yang penting mesti direkodkan di bawah.**
*Clinically significant findings must be recorded below.*

Adalah pesakit sedang atau pernah menghadapi masalah perubatan atau pembedahan pada masa lepas?
*Is subject currently suffering from or has he/she ever suffered in the past from any significant medical or surgical condition?*

☐ **Tidak** *No* ☐ **Ya** *Yes*

**Jika 'Ya', sila nyatakan di bawah satu diagnosis per baris. Sila nyatakan diagnosis dan bukannya gejala.**
*If ''Yes', please list below one diagnosis per line.Please give diagnosis rather than the symptoms.*

| | **Tarikh Permulaan** *Date of onset* | | | **Adakah ia masihsatu malasah yang aktif?** *Is it still an active problem now?* | |
|---|---|---|---|---|---|
| | **Hari** *dd* | **Bulan** *mm* | **Tahun** *yyyy* | **Ya** *Yes* | **Tidak** *No* |
| 1) | | | | ☐ | ☐ |
| 2) | | | | ☐ | ☐ |
| 3) | | | | ☐ | ☐ |

Adakah pesakit pernah mengalami alahan/ kesan sampingan yang teruk daripada mana-mana ubat?
*Has subject ever experienced any allergy or adverse event from any medications?*

☐ **Tidak** *No* ☐ **Ya** *Yes*

**Sila nyatakan *(please specify)*:** ____________________

## 5. **DATA KLINIKAL** *CLINICAL DATA*

**Pengukuran antropometri**
*Anthropometric measurement*

| | | Metric | Percentile/ Z-score |
|---|---|---|---|
| Berat/ w*eight* | : | kg | |
| Tinggi/ *height* | : | cm | |
| Indeks Jisim Tubuh (BMI)/ *Body Mass Index* | : | $kg/m^2$ | |

**Tanda-Tanda Vital**
*Vital signs*

| | Unit *Unit* | Lawatan ke-1 *1st visit* | Lawatan ke-2 *2nd visit* | Lawatan ke-3 *3rd visit* | Lawatan ke-4 *4th visit* |
|---|---|---|---|---|---|
| | | Tarikh *date*: ___ / ___ / ___ | Tarikh *date*: ___ / ___ / ___ | Tarikh *date*: ___ / ___ / ___ | Tarikh *date*: ___ / ___ / ___ |
| Sistolik *Systolic* | mmHg | | | | |
| Diastolik *Diastolic* | mmHg | | | | |
| Nadi *Pulse* | bpm | | | | |
| Suhu badan *Temperature* | °C | | | | |
| Kadar pernafasan *Respiratory rate* | breaths/min | | | | |
| Skala kesakitan (VAS) *Pain score (VAS)* | | | | | |
| Skala kesakitan (Pain Monitor) *Pain score (Pain Monitor)* | | | | | |
| Tandatangan ringkas dan nama pemeriksa *Initials and name of assessors* | | | | | |

***6.*** LOG PENJEJAKAN KESAN SAMPINGAN *ADVERSE EFFECT TRACKING LOG*

| No. | Date Reported | Adverse Event Description | Start Date | End Date | Ongoing (Y or N) | Outcome[1] | Severity/ Grade[2] | Serious (Y or N) | AE Treatment[3] | Expected (Y or N) | Intervention Attribution / Relatedness[5] |
|---|---|---|---|---|---|---|---|---|---|---|---|
| | | | | | | | | | | | |
| | | | | | | | | | | | |
| | | | | | | | | | | | |
| | | | | | | | | | | | |
| | | | | | | | | | | | |
| | | | | | | | | | | | |

**Outcome[1]**
0 – Fatal
1 – Not recovered/not resolved
2 – Recovered w/sequelae
3 – Recovered w/o sequelae
4 – Recovering/Resolving

**Severity/Grade[2]**
1 – Mild
2 – Moderate
3 – Severe
4 – Life-Threatening
5 – Death (Fatal)

**AE Treatment[3]**
0 – None
1 – Medication(s)
2 – Non-medication TX

**Action Taken[4] with Study Intervention**
0 – None
1 – Interrupted
2 – Discontinued
3 – Dose reduced
4 – Dose increased

5 – Not Applicable

**Attribution/ Relatedness[5]**
0 – Definite
1 – Probable
2 – Possible
3 – Unrelated
4 – Not Applicable
(did not receive intervention)

Verified by (prior to data entry)
Name:________________
Date:________________
